# Anti-Inflammatory and Antiplatelet Activities of Plasma Are Conserved Across Twelve Mammalian Species

**DOI:** 10.3390/molecules190811385

**Published:** 2014-07-31

**Authors:** Sagheer Ahmed, Saima Gul, Fazean Idris, Abrar Hussain, Muhammad Zia-Ul-Haq, Hawa Z. E. Jaafar, Marius Moga

**Affiliations:** 1 PAP RSB Institute of Health Sciences, University Brunei Darussalam, Gadong BE1410, Bander Seri Begawan, Brunei Darussalam; E-Mails: sagheer.ahmed@ubd.edu.bn (S.A.); saimaa.gul@gmail.com (S.G.); fazean.idris@ubd.edu.bn (F.I.); 2 Department of Pharmacy, Kohat University of Science & Technology, Kohat 26000, Pakistan; 3 Department of Biotechnology & Informtics, Baluchistan University of Information Technology, Engineering & Management Sciences, Quetta 87300, Pakistan; E-Mail: abrarbangash176@gmail.com; 4 The Patent Office, Karachi 74400, Pakistan; 5 Department of Crop Science, Faculty of Agriculture, University Putra Malaysia, Selangor 43400, Malaysia; 6 Department of Medical and Surgical Specialities, Transilvania University of Brasov, Brasov 500036, Romania; E-Mail: moga.og@gmail.com

**Keywords:** platelet aggregation, arachidonic acid, anti-inflammatory, mammals, evolution

## Abstract

Human plasma inhibits arachidonic acid metabolism and platelet aggregation. This helps human form a haemostatic control system that prevents the progress of certain aggregatory or inflammatory reactions. Whether this property of plasma is unique to human or extends to other species is not well known. It is speculated that this protective ability of plasma remains evolutionarily conserved in different mammals. In order to confirm this, the effect of plasma from 12 different mammalian species was investigated for its inhibitory potential against arachidonic acid metabolism and platelet aggregation. Metabolism of arachidonic acid by cyclooxygenase and lipoxygenase pathways was studies using radio-immuno assay and thin layer chromatography while platelet aggregation in the plasma of various mammals was monitored following turbedmetric method in a dual channel aggregometer. Results indicate that inhibition of AA metabolism and platelet aggregation is a common feature of plasma obtained from different mammalian species, although there exists large interspecies variation. This shows that besides human, other mammals also possess general protective mechanisms against various aggregatory and inflammatory conditions and this anti-inflammatory property of the plasma is evolutionarily conserved in mammalian species. The most likely candidates responsible for these properties of plasma include haptoglobin, albumin and lipoproteins.

## 1. Introduction

Normal hemostasis maintains a fine balance between pro- and antithrombotic activities, during which platelets play a central role [1]. Platelets also play pivotal roles in the activation of immune and inflammatory responses, and in wound healing and repair [2]. Increased platelet activity and aggregation is associated with a wide variety of medical conditions including hypertension, arterial fibrillation, inflammatory bowel disease, rheumatoid arthritis, arteriosclerosis and diabetes [3]. Thromboxane A_2_ (TXA_2_) is one most important proponents of platelet aggregation and is formed by the action of cyclooxygenase (COX) on arachidonic acid (AA). Aspirin- a prototype of non-steroidal anti-inflammatory drugs exert its anti-inflammatory and antiplatelet effects by irreversibly inhibiting COX. Therefore, inhibition of COX and platelet aggregation is an important therapeutic target for treating a number of inflammatory and cardiovascular diseases [4].

Besides drugs available to treat inflammation and platelet aggregation, endogenous mechanisms of our body are also in place to limit unwanted inflammatory reactions. The human blood plasma of adult, but not fetal, inhibits COX-1 *in vitro* [5,6]. Human serum also inhibits the production of prostaglandin (PG) E2, PGF_2α_ and PGI_2_ [7] and since it inhibits COX, it was assumed that serum also inhibits the production of thromboxane (TX) A_2_. This assumption is supported by the finding that human plasma inhibits arachidonic acid (AA)-induced platelet aggregation of washed human platelets suspended in buffer [8]. This shows that humans possess their own defense mechanism against unwanted platelet aggregation. Such observations indicate that adult human plasma or serum inhibits the biosynthesis of AA metabolites formed through the COX enzymatic pathway.

We previously reported that human plasma inhibits platelet aggregation and formation of AA metabolites via lipoxygenase (LOX) pathway [9]. Rat serum also inhibits the production of prostaglandins by leukocytes that are phagocytosing killed bacteria [10]. We recently reported that these activities of plasma are present in the plasma of rabbit with hypercholesterolemia [11]. As a result of these and other studies we know that plasma from human, rat and rabbit possesses activities against AA metabolism and platelet aggregation. However, it is not known whether these properties of plasma are present in other mammals as well. In order to find out whether these activities of plasma are evolutionarily conserved in different mammalian species and to expand our knowledge about effects of plasma in different mammalian species, we investigated the plasma obtained from 12 mammalian species on COX and LOX enzymatic pathways of AA metabolism and platelet aggregation.

## 2. Results and Discussion

Various stimuli activate phospholipase A_2_ (PLA_2_) which in turn releases AA from membrane phospholipids. Prostaglandins and leukotrienes (both pro-inflammatory mediators) are derived from AA through the actions of two major enzymes COX and LOX respectively [12]. Therefore inhibition of these two enzymes, especially inducible form of COX (COX-2), is an important therapeutic target [13,14]. Furthermore, inhibition of COX and LOX by plasma lipoproteins points to an important regulatory link in the body’s defense system [11]. Our previous work shows that AA metabolism and human platelet aggregation is inhibited by human plasma, serum and other body fluids that this action of plasma is related to human hemoglobin and haptoglobin [15,16]. In the present study we found that plasma from different mammalian species is capable of inhibiting the cyclooxygenation and lipoxygenation of AA.

Results of this study indicate that plasma obtained from all 12 mammalian species inhibited AA metabolites formed through COX pathway as well as LOX pathway with high inter-species variation (Table 1). Plasma concentrations which exhibited 50% inhibition of platelet aggregation (IC_50_) showed large variations amongst the 12 mammalian species and results in some interesting inter-species and intra-species correlations related to their effects on AA metabolism and platelet aggregation.

**Table 1:** Comparative effects of plasma obtained from different mammalian species on cyclooxygenase and lipoxygenase mediated pathways of platelet arachidonic acid metabolism.

Plasma	IC_50_ % v/v (Mean ± SEM)		
	LP1	12-HETE	TXB_2_
Human (*Homo sapiens*)	0.5 ± 0.07	0.8 ± 0.05	0.5 ± 0.03
Baboon (*Papio hamadryas*)	15.0 ± 3.0 ^*^	12.0 ± 4.0 ^*^	7.5 ± 2.0 ^*^
Bovine (*Bison bison*)	0.5 ± 0.06	0.5 ± 0.03 ^*^	1.0 ± 0.20 ^*^
Calf (*Bison bison*)	1.0 ± 0.50 ^*^	1.20 ± 0.3 ^*^	15 ± 0.40 ^*^
Cat (*Felis catus*)	7.0 ± 2.0 ^*^	5.0 ± 1.0 ^*^	2.5 ± 0.80 ^*^
Dog (*Canis lupus familiaris*)	2.0 ± 0.8 ^*^	6.5 ± 1.20 ^*^	1.0 ± 0.25 ^*^
Goat (*Capra aegagrus hircus*)	0.3 ± 0.09 ^*^	2.5 ± 1.0 ^*^	2.3 ± 1.0 ^*^
Horse (*Equus caballus*)	15.0 ± 4.0 ^*^	12 ± 2.5 ^*^	15.0 ± 4.0 ^*^
Pig (*Sus barbatus*)	15.0 ± 4.0 ^*^	Stimulant	7.2 ± 2.0 ^*^
Rabbit (*Oryctolagus cuniculus*)	4.0 ± 1.0 ^*^	7.5 ± 1.50 ^*^	2.5 ± 1.0 ^*^
Rat (*Rattus rattus*)	1.50 ± 0.25 ^*^	2.5 ± 0.70 ^*^	1.30 ± 0.10 ^*^
Sheep (*Ovis aries)*	2.50 ± 0.10 ^*^	1.5 ± 0.25 ^*^	2.5 ± 0.70 ^*^
Chicken (*Gallus gallus*)	5.0 ± 1.50 ^*^	6.8 ± 1.70 ^*^	5.0 ± 2.0 ^*^

^*^ p < 0.05 compared to the effect of human plasma, n = 5–7.

**Figure 1:**
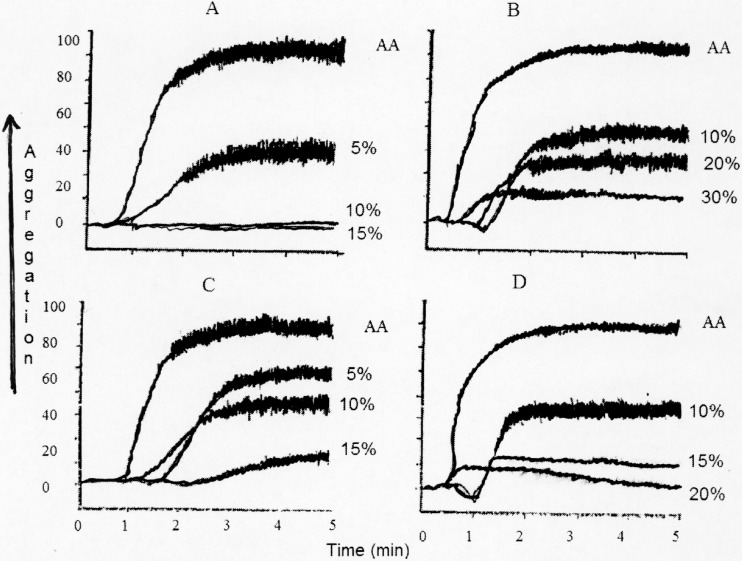
Effects of plasma from cat (**A**), baboon (**B**), bovine (**C**) and dog (**D**) on AA-induced platelet aggregation. N (4).

The two primates, human and baboon showed large interspecies dose variation against AA metabolism and platelet aggregation. LOX product 12-HETE was inhibited by baboon plasma at 15-times and LP1 at 30-times higher concentrations as compared to human indicating greater potency of human plasma (Table 1). Compared to human, baboon plasma inhibited COX metabolite TXB_2_ at 15-times higher concentration which points to lesser activity of baboon plasma against COX enzyme. It was therefore, expected that baboon plasma would not be able to inhibit platelet aggregation or would do so at many fold higher plasma concentrations compared to human. However, IC_50_ values for baboon were just 3-times higher against platelet aggregation when compared with human (Table 2 and Figure 1). This suggests that the contribution of TXB_2_ inhibitory constituents in inhibiting platelet aggregation is less in baboon plasma compared to human.

Among the mammals of the artiodactyla order, cattle, calf, sheep and goat inhibited AA metabolism at roughly same IC_50_ values as humans while pig showed inhibition of LP1 at 30-times higher concentration compared to both human and cattle (Table 1). Against TXB_2_, IC_50_ of pig plasma was 14-times higher than human and 7-times higher than cattle. However, pig plasma stimulated the formation of 12-HETE and this property was only observed in pig plasma among the 12 species. Calf plasma inhibited LOX products at twice the plasma concentration of human and cattle. Surprisingly 40- and 20-times higher plasma concentrations were required to inhibit COX metabolite (TXB_2_), compared to human and cattle respectively. Platelet aggregation was not inhibited by calf plasma up to a concentration of 25% (Table 2). Since calf plasma inhibited TXB_2_, albeit at extraordinarily high concentrations, but did not prevent platelet aggregation, it seems that TXB_2_ has little role in aggregating platelets in calf. This effect is similar to human fetal plasma which does not inhibit platelet aggregation. There seems to be a similar correlation between calf and cattle plasma as observed between human and fetal plasma in inhibiting platelet aggregation. However, fetal plasma is also ineffective against AA metabolism while calf serum possesses inhibitory activity against AA metabolism.

In the mammals of carnivore order, cat plasma inhibited LOX product LP1 at 14-times higher concentration while 12-HETE was inhibited at 6-times higher concentrations compared to human. Cat plasma inhibited TXB_2_ at 5-times higher concentrations compared to human. IC_50_ values for cat plasma against platelet aggregation was similar to human plasma (Figure 1). Dog plasma inhibited LP1 at 4-times, 12-HETE at 8-times and TXB_2_ at 2-times higher concentrations compared to human. Platelet aggregation was inhibited by dog plasma at twice the concentrations required by human (Figure 1) plasma.

**Table 2:** Comparative effects of plasma obtained from different species on AA induced human platelet aggregation.

Plasma	IC_50_ % v/v (Mean ± SEM)
Human	4.2 ± 0.85
Baboon	12.0 ± 1.65 ^*^
Bovine	9.6 ± 0.40 ^*^
Calf	NA
Cat	4.0 ± 0.63
Dog	9.0 ± 1.50 ^*^
Goat	4.6 ± 0.76
Horse	2.8 ± 0.64 ^*^
Pig	14.0 ± 2.09 ^*^
Rabbit	5.5 ± 0.42 ^*^
Rat	11.0 ± 2.00 ^*^
Sheep	13.1 ± 1.90 ^*^
Chicken	NA

^*^ *p* < 0.05 compared to the effect of human plasma, n = 5–7. NA: Not Applicable.

**Figure 2:**
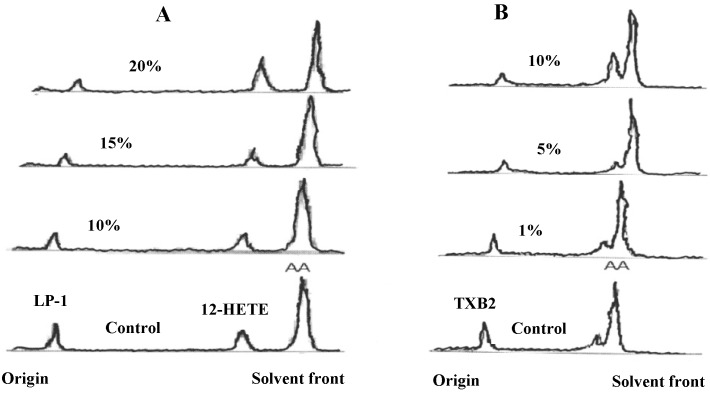
Effects of pig (*Sus barbatus*) plasma on COX and LOX medicated metabolism of AA. N(4).

Rabbit plasma inhibited LP1 at 8-times, 12-HETE at 9-times and TXB2 at 5-times higher concentrations compared to human plasma while IC50 values against platelet aggregation were only slightly higher. Rat plasma inhibited LP1, 12-HETE and TXB_2_ and platelet aggregation at roughly 3-times higher concentrations compared to human plasma. Horse displayed inhibitory properties against LP1 and TXB_2_ at 30-times and against 12-HETE at 15-times higher concentrations while against platelet aggregation, the IC_50_ was one half to that of human. Pig plasma inhibited platelet aggregation, LP-1 and TXB_2_ but stimulated 12-HETE production (Table 1 and Figure 2). Chicken plasma, although inhibited AA metabolism through both COX and LOX, was unable to inhibit AA-induced platelet aggregation (Table 1 and Table 2). This suggests the presence of stimulatory components in chicken plasma dominant to inhibitory constituents.

Although our study does not identify the plasma constituents responsible for observed activities, a number of potential candidates can be identified based on previous studies. The plasma constituent(s) most likely responsible for inhibition of AA metabolism and platelet aggregation include plasma albumin, haptoglobin and lipoproteins. While albumin is found in all mammals and has been shown to possess inhibitory activities against AA metabolism and platelet aggregation in human, studies show it is only partly responsible for these effects of plasma [9,17]. Previous work from our group has shown that human haptoglobin also possesses significant inhibitory activity against AA metabolism and platelet aggregation [5,8,9,15,16,17]. In healthy individuals, the albumin concentration in plasma is around 35–55 mg/mL [18] while the concentration of haptoglobin is roughly 100-times lower than that of albumin [19]. However, haptoglobin is known to be more potent inhibitor of prostaglandin synthesis than albumin [9]. The IC_50_ of pure haptoglobin against prostaglandin synthesis is about 100 µg [5] while in the current study human plasma displayed IC_50_ of 0.5% against prostaglandin synthesis which corresponds to about 50µg of haptoglobin. This indicates that constituents of plasma other than haptoglobin are also involved, including albumin as stated above.

However, unlike albumin which is found in all vertebrates, haptoglobin is found only in mammals and in bony fish but not in chicken [20]. This may account for the observed ineffectiveness of the chicken plasma against platelet aggregation in the current study. Inhibition of the prostaglandin synthesis by human haptoglobin has been reported previously, albeit in the presence of reduced glutathione [21], and by haptoglobin and plasma of rats with inflammation [22]. This function of haptoglobin was also confirmed in rabbits in a study which shows a direct relationship between AA-induced hypotension in rabbits and haptoglobin levels [23].

Another constituent of plasma that may be responsible, in part, for the observed actions in our study are plasma lipoproteins. However, the role of plasma lipoproteins in inhibiting AA metabolism and platelet aggregation remains controversial. Several *in vitro* [24,25] and *in vivo* [26,27] studies showed that LDL directly causes an increase in platelet aggregation and TXA_2_ release, which could contribute to intravascular thrombus formation and vasoconstriction. Previous work from our lab has shown that LDL and HDL obtained from hypercholesterolemic rabbits inhibit *in vitro* AA metabolism and platelet aggregation while VLDL was ineffective [11]. Therefore, it is also likely that intra and inter species differences in IC_50_ values in the current study may be due to a difference in the concentrations of plasma proteins responsible for the inhibition of AA metabolism and platelet aggregation.

These properties of plasma indicate that an important function of plasma may be concerned with the endogenous control of AA metabolism through COX and LOX enzymes. The circumstances in which plasma may act in this way in the body remains to be determined: but it probably forms a part of a normal haemostasis and keeps check on inflammatory reactions. Further work in this area will provide useful information about natural regulatory mechanisms in diseases associated with imbalanced prostaglandin production and also lead to developments of suitable methods for detecting types of anti-inflammatory activity more relevant to human disease. In light of the above, the determination of the exact concentrations and identities of the plasma constituents responsible for the observed effects in different mammalian species is important. Such comparative studies would be important in relating the differential effects of mammalian plasma on AA metabolism and platelet aggregation.

## 3. Experimental Section

### 3.1. Sample Collection

Blood collection from animals was performed using the appropriate restraints such as physical restraints (including restraint box) or chemical restraint (e.g., anesthesia in chicken). Blood was withdrawn by primary care veterinarian observing all the necessary precautions required for such procedures. Blood was obtained from the cubital vein in human, saphenous or cephalic vein after administration of anesthesia in baboon, from the marginal ear vein in rabbits, from tail in rats, directly from heart in anesthetized chicken while jugular vein was used to obtain blood from the rest of the species. The study was approved by the “Animal Committee” of the department of Pharmacy, Kohat University of Science & Technology, Pakistan. The experiments were conducted in accordance with institutional guidelines, which are in compliance with the *Guide for the Care and the Use of Laboratory Animals* published by the National Institutes of Health [28].

### 3.2. Measurement of Platelet Aggregation

Platelet aggregation was measured by slight modification of previously described method [29,30,31]. Plasma obtained from a species was mixed with 10 units/mL of heparin and centrifuged at 200 g for 15 min. The platelet rich plasma (PRP) thus obtained was centrifuged at 600 g for 15 min and the resulting platelet pellet was washed with saline. The pellet of washed platelets was then suspended in 0.5 volumes of 0.1 mol/L Tris-HCI buffer, pH 7.4. Platelet aggregation was monitored with a Chronolog aggregometer (Chrono-Log Corporation, Big Lake, MN, USA). Test plasma was added to 0.5 mL of platelet suspension at 37 °C for 5 min, prior to the addition of sodium arachidonate (aggregation agent). The percentage inhibition due to test plasma compared with control (AA alone) was calculated at 1, 2, 3 and 4 min after addition of AA [32].

### 3.3. AA Metabolism by Human Platelets

AA metabolism was studied using the methods as described previously [33,34]. In brief plastic bags containing 30–40 mL concentrated PRP were obtained from different species. Phosphate buffer was used to wash the sedimented platelets twice after centrifuging the PRP for twenty minutes at 1200 g. The buffer containing EDTA (0.2 mM) and NaCl (0.15 M) had a PH of 7.4. After washing, platelets were again suspended in the same buffer but without EDTA. Using a polytron homogenizer (KINEMATICA, INC., Bohemia, NY, USA) this suspension was homogenized for 15 s at 4 °C. The homogenate was again centrifuged at 1200 g for twenty minutes. After centrifugation, the supernatant was isolated and incubated with 10 µg unlabeled AA and 0.1 µCi [1-^14^C] in the presence and absence of test plasma. The reaction was allowed to proceed with gentle shaking at 37 °C for 15 min. The reaction was stopped by adding ethyl acetate (7 mL) and citric acid (0.4 mL of 0.4 M). Again centrifugation was done at 4 °C to separate the organic layer. This organic layer was evaporated to dryness using liquid nitrogen. The residual material was applied (20 µL) to thin layer chromatography plates containing silica gel. The standards of all the metabolites such as 12-hydroxy-eicosatetraenoic acid (HETE)-a stable metabolite of 12-HpETE, lipoxygenase product 1 (LP1), TXB_2_ (a more stable metabolite of TXA_2_), and AA were separately plotted. The plates were developed in two different solvent systems. Various lipoxygenasse products such as 12-HETE were separated using acetic acid/ether/petroleum ether (1:50:50, v/v) solvent system while ethyl acetate/iso-octane/water/acetic acid (11:5:10:2, v/v upper phase) solvent system was used to separate cyclooxygenase products. Finally radioactive zones were located and quantified.

### 3.4. Statistical Analysis

Data are represented as the mean ± SEM unless stated otherwise. Statistical significance was assessed by student t test. *p* < 0.05 was considered statistically significant.

## 4. Conclusions

In conclusion, the conservation of antiplatelet and dual LOX and COX inhibitory activities of plasma across various mammalian species indicates that this phenomenon probably forms a part of a haemostatic control system that could help to curtail the progress of certain aggregatory or inflammatory reactions. The fact that these anti-inflammatory activities of plasma are conserved across various species also suggests that there might be some common plasma components responsible for these observed effects. The most important candidates for these unique properties of plasma are albumin, haptoglobin and plasma lipoproteins.
